# Ressuscitação Cardiopulmonar Extracorpórea para Parada Cardiorrespiratória Intra-Hospitalar: Experiência de um Centro na América Latina

**DOI:** 10.36660/abc.20240820

**Published:** 2026-04-14

**Authors:** Lucrecia M. Burgos, Leonardo A. Seoane, Ana Spaccavento, Juan F. Furmento, Juan P. Costabel, Rocio C. Baro Vila, Maria A. de Bortoli, Daniel O. Navia, Fernando Piccinini, Mariano Vrancic, Mirta Diez

**Affiliations:** 1 Instituto Cardiovascular de Buenos Aires Pulmonary Hypertension and Transplant Department Buenos Aires Argentina Instituto Cardiovascular de Buenos Aires (ICBA), Heart Failure, Pulmonary Hypertension and Transplant Department, Buenos Aires – Argentina; 2 Instituto Cardiovascular de Buenos Aires Critical Care Cardiology Department Buenos Aires Argentina Instituto Cardiovascular de Buenos Aires (ICBA), Critical Care Cardiology Department, Buenos Aires – Argentina; 3 Instituto Cardiovascular de Buenos Aires Cardiac Surgery Department Buenos Aires Argentina Instituto Cardiovascular de Buenos Aires (ICBA), Cardiac Surgery Department, Buenos Aires – Argentina

**Keywords:** Parada Cardíaca, Oxigenação por Membrana Extracorpórea, Reanimação Cardiopulmonar

## Abstract

**Fundamento:**

A ressuscitação cardiopulmonar extracorpórea (ECPR) consiste na utilização de oxigenação por membrana extracorpórea (ECMO) em pacientes nos quais as medidas convencionais de ressuscitação cardiopulmonar não obtiveram retorno sustentado da circulação espontânea após parada cardiorrespiratória. As informações disponíveis em centros na América Latina são limitadas.

**Objetivo:**

Avaliar os desfechos clínicos intra-hospitalares e de longo prazo de pacientes adultos tratados com ECPR após parada cardiorrespiratória (PCR) intra-hospitalar em um centro de alta complexidade na Argentina

**Métodos:**

Foi realizado um estudo de coorte unicêntrico. Pacientes adultos consecutivos submetidos a ECPR entre 2014 e 2023 foram analisados retrospectivamente. Foram incluídos casos de PCR intra-hospitalar presenciados e de provável origem cardíaca.

**Resultados:**

Foram incluídos 15 pacientes na análise, representando 16% dos casos de ECMO venoarterial implantados no centro durante esse período. A mediana de idade foi de 46 anos (intervalo interquartil [IIQ]: 34 a 57), sendo 33% do sexo feminino. A canulação foi periférica em 93,3% dos casos e 66% dos procedimentos de ECMO foram realizados com uma estratégia inicial de ponte para recuperação. A duração mediana do suporte circulatório foi de 5 dias (IIQ: 2 a 9). A ECMO venoarterial foi removida com sucesso em 60% dos pacientes e a sobrevida hospitalar foi de 46%. O acompanhamento mediano após a alta foi de 12 meses (IIQ: 3 a 34), com 100% de sobrevida em longo prazo.

**Conclusão:**

A ECMO venoarterial, como tratamento para PCR intra-hospitalar em nosso centro, demonstrou sobrevida até a alta hospitalar e sobrevida em longo prazo aceitáveis. A ECMO venoarterial pode ser um tratamento eficaz em pacientes criteriosamente selecionados quando as terapias convencionais falham e pode ser útil e aplicável em países de baixa e média renda, onde o acesso a outros dispositivos de assistência circulatória é limitado.

## Graphical abstract

**Figura Central:**
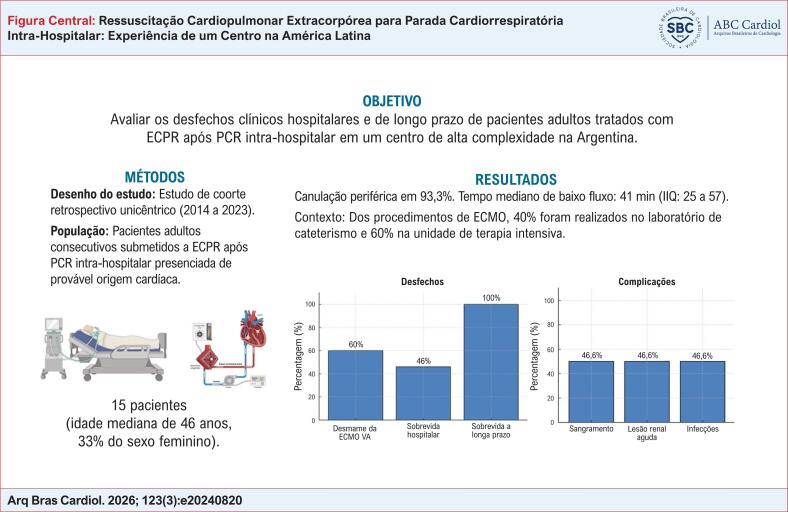
Ressuscitação Cardiopulmonar Extracorpórea para Parada Cardiorrespiratória Intra-Hospitalar: Experiência de um Centro na América Latina

ECMO: oxigenação por membrana extracorpórea; ECPR: ressuscitação cardiopulmonar extracorpórea; IIQ: intervalo interquartil; PCR: parada cardiorrespiratória; VA: venoarterial. Gerado com https://BioRender.com.

## Introdução

A parada cardiorrespiratória (PCR) intra-hospitalar é um evento agudo que pode afetar qualquer paciente hospitalizado. A PCR é uma das principais causas de morbimortalidade em todo o mundo.^1–4^ Apesar dos avanços significativos na administração de ventilação mecânica, medicamentos, compressões torácicas e desfibrilação elétrica ao longo dos últimos 50 anos, alcançar o retorno da circulação espontânea (RCE) por meio da ressuscitação cardiopulmonar (RCP) convencional ainda representa um desafio. Além disso, mesmo após a obtenção do RCE, alguns pacientes podem apresentar nova PCR e/ou desfechos neurológicos desfavoráveis.^5,6^

A ressuscitação cardiopulmonar extracorpórea (ECPR, do inglês, *extracorporeal cardiopulmonary resuscitation*) consiste na utilização de oxigenação por membrana extracorpórea (ECMO, do inglês, *extracorporeal membrane oxygenation*) em pacientes nos quais as medidas convencionais de RCP falharam em alcançar o RCE sustentado após uma PCR.^i^ A ECPR pode ser considerada quando o tempo sem circulação espontânea é curto e as tentativas de ressuscitação são adequadas (ou seja, RCP > 10 minutos, uso apropriado de medicamentos guiados por diretrizes, como epinefrina e vasopressina, e desfibrilação para ritmos chocáveis).^1–4^

A ECPR é cada vez mais utilizada em todo o mundo como técnica de resgate em pacientes com PCR refratária.^7^ Embora atualmente não existam recomendações sistemáticas para a indicação de ECMO nesse contexto, ela pode ser considerada como uma terapia emergencial quando a RCP convencional falha em casos selecionados.^8^ Atualmente, não há relatos de ensaios clínicos randomizados controlados comparando os resultados da ECPR com a RCP convencional em PCR intra-hospitalar.^4^ Em contrapartida, existem alguns ensaios clínicos em PCR extra-hospitalar, com resultados heterogêneos.^9–11^

Estudos de coorte pareados por propensão relataram que os desfechos associados ao uso de ECMO são superiores aos da RCP convencional isolada em pacientes com PCR intra-hospitalar.^12–14^ Além disso, uma metanálise recente constatou que a ECPR pode reduzir a mortalidade intra-hospitalar.^15^ No entanto, há pouca informação disponível em países de baixa e média renda. As barreiras à sua aplicação incluem acessibilidade, falta de recursos, potenciais complicações e a necessidade de uma equipe especializada para implantação e manejo adequados.

O objetivo do presente estudo foi analisar e relatar as características e os desfechos clínicos de uma coorte consecutiva de pacientes adultos tratados com ECPR após uma PCR intra-hospitalar em um centro de alta complexidade na Argentina.

## Materiais e métodos

Foi realizado um estudo de coorte unicêntrico. Relatamos as características e os desfechos clínicos de uma coorte consecutiva de pacientes adultos tratados com ECPR após PCR intra-hospitalar em um centro de alta complexidade na Argentina.

### Pacientes

Foram analisados pacientes adultos consecutivos submetidos a ECPR entre janeiro de 2014 e julho de 2023. Em nosso hospital, os critérios de inclusão para ECPR foram: idade entre 18 e 70 anos, PCR intra-hospitalar presenciada e provável origem cardíaca (principalmente taquicardia ventricular ou fibrilação ventricular como ritmo inicial). A ausência de comorbidades limitantes da vida previamente conhecidas (doença pulmonar obstrutiva crônica, insuficiência renal terminal, insuficiência hepática, doença terminal), ausência de dissecção aórtica e doença arterial periférica grave, e tempo estimado entre a PCR e o fluxo de ECMO < 60 minutos foram critérios adicionais para inclusão no presente estudo. Pacientes que receberam suporte de ECMO para choque cardiogênico e posteriormente apresentaram PCR foram excluídos da análise.

### Descrição da equipe e do centro de ECMO

A equipe de ECMO, embora faça parte da clínica de transplante e assistência ventricular, é composta por especialistas de diversos serviços: cirurgiões cardiovasculares, cardiologistas de terapia intensiva, especialistas em insuficiência cardíaca, perfusionistas, enfermeiros, cinesiologistas, cardiologistas especializados em ultrassom, anestesiologistas, cardiologistas intervencionistas, nutricionistas, hematologistas e infectologistas. A equipe de ECMO é um componente da equipe de choque, focada especificamente na ECMO venoarterial como suporte circulatório. Os principais membros da equipe (cirurgiões cardiovasculares, perfusionistas, cardiologistas e enfermeiros) devem estar disponíveis 24 horas por dia, 365 dias por ano, para canulação de emergência ou para tratar prontamente complicações da ECMO. Complicações relacionadas à canulação, como lesão vascular e isquemia de membros, foram prevenidas pelo uso rotineiro de cateteres de perfusão distal. A canulação para ECMO foi realizada por cardiologistas intervencionistas ou cirurgiões cardiovasculares, dependendo do contexto.

Os principais objetivos da criação de uma equipe de ECMO foram padronizar os processos, determinar critérios unificados de inclusão e exclusão para o implante de ECMO venoarterial e garantir assistência ventricular oportuna em casos de choque cardiogênico ou PCR refratários. Após o implante, a equipe é responsável pelo monitoramento e acompanhamento diário do paciente, determinando o momento e o modo de desmame da assistência. O foco inicial foi a criação de listas de verificação para montagem, purga e implante da ECMO, bem como protocolos de ativação da equipe. Posteriormente, foram desenvolvidos protocolos para implante percutâneo e cirúrgico, manejo da anticoagulação, utilização de ultrassom (para implante, monitoramento e desmame), profilaxia de infecções, nutrição e cuidados de enfermagem.

A criação da equipe de ECMO, além da organização e padronização dos processos, envolveu aprendizado teórico e treinamento de habilidades psicofísicas.

Todos os membros principais da equipe de ECMO completam um programa de treinamento institucional estruturado, incluindo exercícios de simulação de alta fidelidade realizados pelo menos duas vezes por ano, com o objetivo de manter a proficiência técnica e a coordenação da equipe. Desde 2021, nosso centro é certificado pela Extracorporeal Life Support Organization (ELSO). Segundo dados da ELSO, o volume médio anual de ECMO venoarterial em adultos em centros certificados da América Latina em 2022 foi de 5 a 6 sessões.^16^ Atualmente, nosso centro tem um volume médio de 15 procedimentos de ECMO venoarterial por ano.

Nossa instituição aplica protocolos de não ressuscitar sob supervisão ética padronizada, principalmente em pacientes com doença em estágio terminal ou diretivas antecipadas de vontade.

### Suporte ECMO e manejo pós-parada cardiorrespiratória

A equipe de ECMO era acionada em pacientes com PCR refratária que não obtiveram RCE após 10 minutos de RCP convencional. No caso de implantação da ECMO no laboratório de cateterismo, a punção e a canulação foram iniciadas por cardiologistas intervencionistas, enquanto o procedimento foi conduzido por cirurgiões cardiovasculares em pacientes na unidade de terapia intensiva. A Tabela 1 descreve o estabelecimento do suporte ECMO e o manejo pós-PCR.

**Tabela 1:** Estabelecimento de suporte ECMO e manejo dos cuidados pós-parada cardiorrespiratória

Após a inserção da cânula: – Eliminar todo o ar do circuito. Verificar a presença de ar ou bolhas no circuito, na membrana ou nas conexões. – Aumentar as RPM e remover as pinças do circuito. – O fluxo sanguíneo da ECMO venoarterial deve ser ≥3 L/min. Interromper as compressões mecânicas. As infusões de vasopressores e inotrópicos podem precisar ser reduzidas rapidamente para atingir uma PAM ≥ 60 mmHg para pressão de perfusão orgânica e < 80 mmHg para minimizar o risco de distensão do ventrículo esquerdo.
Confirmar a posição da cânula com fluoroscopia ou ecocardiografia antes de fixá-la e realizar o curativo.
Medir a PAM com uma linha arterial no membro superior direito (radial ou braquial).
Ajustar o fluxo de gás de varredura da ventilação mecânica para evitar hipocapnia e hiperóxia.
Corrigir a possível insuficiência de drenagem com fluidos/transfusão/redução gradual do fluxo sanguíneo da ECMO venoarterial, caso exceda a necessidade; a circulação total é composta pelo débito cardíaco nativo, se presente, mais o fluxo sanguíneo da ECMO venoarterial.
Garantir o posicionamento correto do tubo endotraqueal e do cateter venoso central.
Sedação e analgesia.
Controle da temperatura alvo: 33 a 36 °C por 24 horas
Realizar ultrassonografia à beira do leito para identificar possíveis complicações e avaliar a competência valvar e a distensão do ventrículo esquerdo.

ECMO: oxigenação por membrana extracorpórea; PAM: pressão arterial média; RPM: rotações por minuto. Adaptado de Richardson et al.^8^

A massagem cardíaca externa foi mantida durante toda a fase de canulação. Utilizamos uma técnica de Seldinger modificada para canular a veia femoral e a artéria femoral com cânulas de 19-25 Fr e 15-21 Fr, respectivamente. O fluxo sanguíneo da ECMO foi ajustado para atingir uma meta de 2,5 L/min/m^2^, de acordo com as necessidades metabólicas dos pacientes. O fluxo de gás da ECMO foi ajustado para manter uma pCO_2_ arterial de aproximadamente 40 a 45 mmHg. A infusão de heparina foi utilizada para anticoagulação, a fim de manter o tempo de coagulação ativada entre 160 e 180 segundos. O alvo de hematócrito era de 30% a 35%. Um cateter percutâneo adicional de 7-9 Fr foi colocado distalmente à cânula arterial da ECMO na artéria femoral para prevenir isquemia de membro inferior.^17^ Foi realizada ecocardiografia diária. O desmame da ECMO era considerado pelo menos 24 a 48 horas após o RCE, seguindo o mesmo protocolo em cada paciente.^18,19^

A ECMO venoarterial central foi inserida sob anestesia geral por meio de uma incisão de esternotomia, implantando uma cânula na aorta e a outra na veia cava superior.

### Desfechos

O desfecho primário foi a sobrevida hospitalar. Os desfechos secundários incluíram o desmame da ECMO venoarterial, a sobrevida condicional em longo prazo, o estado neurológico na alta hospitalar, eventos hemorrágicos e trombóticos, necessidade de terapia renal substitutiva e complicações vasculares. O estado neurológico foi avaliado de acordo com a categoria de desempenho cerebral (*cerebral performance category* – CPC). As categorias de desempenho foram definidas da seguinte forma: CPC 1, consciente e alerta com função normal ou apenas leve incapacidade; CPC 2, consciente e alerta com incapacidade moderada; CPC 3, consciente com incapacidade grave; CPC 4, em coma ou em estado vegetativo persistente; e CPC 5, morte cerebral certificada ou óbito pelos critérios tradicionais. Um desfecho neurológico favorável foi definido como uma pontuação de 1 ou 2.^20^

### Estratégias de ponte durante o suporte ECMO

Como parte do padrão de atendimento do centro e dos protocolos da equipe de ECMO, ao instalar a ECMO venoarterial, uma ponte inicial é estabelecida e o dispositivo de suporte circulatório mecânico (SCM) é implantado conforme a finalidade clínica.^21,22^ Para pacientes que apresentaram PCR sem histórico cardiovascular prévio, a ponte inicial visa a recuperação dos órgãos. As pontes e as características que orientam sua respectiva seleção são descritas a seguir:

– Ponte para decisão: O SCM é indicado para manter o paciente vivo em casos de colapso circulatório agudo refratário ao tratamento farmacológico, com risco imediato de morte, até que uma avaliação abrangente da condição e opções terapêuticas possa ser realizada.
– Ponte para elegibilidade: Visa melhorar a função de órgãos danificados por baixo débito cardíaco ou para melhorar condições clínicas que aumentam o risco de transplante, como hipertensão pulmonar ou caquexia cardíaca, entre outras. O uso do SCM permite que o paciente se torne um candidato a transplante cardíaco.
– Ponte para transplante: É indicada como suporte de vida para pacientes de alto risco na lista de espera até que um órgão esteja disponível.
– Ponte para recuperação: O SCM permite que o paciente seja mantido vivo até que sua função cardíaca melhore o suficiente para a explantação. É utilizado em situações clínicas onde se presume melhora, como miocardite aguda, infarto agudo do miocárdio, cardiomiopatia periparto, entre outras.
– Terapia de destino: Uso a longo prazo de SCM como alternativa ao transplante cardíaco em pacientes que não são candidatos ao transplante.

### Coleta de dados e acompanhamento

Os dados demográficos e clínicos basais, comorbidades, exame físico na admissão (frequência cardíaca, pressão arterial sistólica e diastólica), apresentação clínica, resultados laboratoriais, tratamento médico prévio e terapia implementada para cada paciente foram coletados em um banco de dados específico do centro. O acompanhamento foi realizado por meio da revisão de prontuários médicos e contato telefônico foi estabelecido para pacientes sem acompanhamento.

### Análise estatística

As variáveis contínuas foram expressas como média e desvio padrão ou mediana e intervalo interquartil (IIQ), de acordo com a distribuição observada. Foram utilizados os testes de Kolmogorov-Smirnov ou Shapiro-Wilk dependendo do tamanho da amostra e das características da distribuição. As variáveis categóricas foram expressas em números e porcentagens. O SPSS Statistics, versão 23.0 (IBM Corporation, Armonk, NY) foi utilizado para a análise estatística.

### Considerações éticas

O presente estudo foi aprovado pelo comitê de ética em pesquisa institucional e registrado na plataforma PRIISA.BA da Secretaria de Saúde da Cidade de Buenos Aires. No momento da internação, os pacientes assinaram um termo de consentimento para a transferência de seus dados pessoais para fins científicos. O estudo foi realizado em conformidade com as normas nacionais e internacionais de proteção de participantes de pesquisa, incluindo a Declaração de Helsinque, a Resolução n° 1480/2011 do Ministério da Saúde Nacional, a Lei n° 3301 da Cidade de Buenos Aires, a Resolução n° 6677/10 da ANMAT e suas emendas 4008 e 4009.

## Resultados

Foram incluídos 15 pacientes na análise, representando 16% dos procedimentos de ECMO venoarterial implantados no centro durante esse período. Dos procedimentos, 40% foram realizados no laboratório de cateterismo e o restante na unidade de terapia intensiva (Figura Central).

A mediana da idade foi de 46 anos (IIQ: 34 a 57), sendo 33% do sexo feminino. Nenhum paciente tinha histórico de doença pulmonar obstrutiva crônica, doença renal crônica, acidente vascular cerebral ou anemia. As principais etiologias subjacentes foram: síndrome coronariana aguda (53,3%), tempestade elétrica em cardiomiopatia arritmogênica (6,7%), cardiomiopatia idiopática (13,4%), cardiomiopatia restritiva idiopática (6,7%) e miocardite (6,7%). As demais características basais estão descritas na Tabela 2. Todos os pacientes (100%) apresentavam ritmo primário chocável.

**Tabela 2:** Características basais

Variáveis	n = 15
Idade, anos, mediana (IIQ)	46 (34-57)
Hipertensão arterial	5 (33,3%)
Dislipidemia	5 (33,3%)
Diabetes	1 (6,7%)
Tabagismo	2 (13,3%)
Doença renal crônica	0 (0%)
Fibrilação atrial	2 (13,3%)
Doença arterial coronariana	1 (6,7%)
Acidente vascular cerebral	0 (0%)
Doença arterial periférica	1 (6,7%)
Anemia	0 (0%)
DPOC	0 (0%)

DPOC: doença pulmonar obstrutiva crônica; IIQ: intervalo interquartil.

A canulação foi periférica em 93,3% dos casos e 66% dos procedimentos de ECMO foram iniciados com uma estratégia inicial de ponte para recuperação. O tempo mediano de baixo fluxo (intervalo entre a PCR e o início da ECMO) foi de 41 minutos (IIQ: 25 a 57). Em relação às estratégias de descompressão do ventrículo esquerdo, 66,6% necessitaram de balão intra-aórtico; 2 pacientes necessitaram de descompressão cirúrgica, e 2 pacientes foram submetidos à descompressão percutânea.

A duração mediana do suporte circulatório foi de 5 dias (IIQ: 2 a 9), e a mediana do tempo de internação foi de 14 dias (IIQ: 11 a 21).

**Figura 1:**
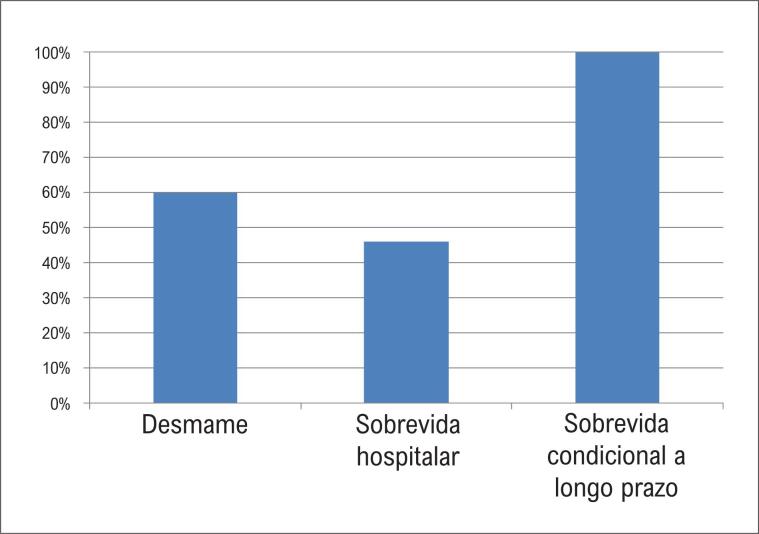
Sobrevida hospitalar e sobrevida condicional a longo prazo.

A ECMO venoarterial foi removida com sucesso em 60% dos pacientes e a sobrevida hospitalar foi de 46% (Figura 1). Um paciente necessitou de transplante cardíaco de emergência.

As complicações mais comuns durante o suporte com ECMO venoarterial foram sangramento (46,6%), infecções (46,6%), lesão renal aguda (46,6%) e ventilação mecânica prolongada com necessidade de traqueostomia (26,6%), conforme demonstrado na Tabela 3.

**Tabela 3:** Complicações da oxigenação por membrana extracorpórea

Complicação	N = 15
Hemorragia, n (%)	7 (46,6%)
Lesão renal aguda, n (%)	7 (46,6%)
Infecções, n (%)	7 (46,6%)
Traqueostomia, n (%)	4 (26,6%)
Arritmias, n (%)	6 (40,0%)
Tamponamento, n (%)	2 (13,3%)
Isquemia de membro inferior, n (%)	4 (26,6%)
Acidente vascular cerebral, n (%)	2 (13,3%)
Convulsões, n (%)	2 (13,3%)
Morte encefálica, n (%)	0 (0%)

O sangramento mais frequente ocorreu no local da canulação, em 4 pacientes (26,6%), seguido por tamponamento cardíaco (13,3%). Apenas um paciente apresentou sangramento gastrointestinal (6,6%). Embora a insuficiência renal tenha ocorrido em 46,6% dos casos, apenas 4 pacientes necessitaram de hemodiálise (26,6%).

Em relação às infecções, o microrganismo mais frequentemente isolado foi *Klebsiella* sp. (13,3%). Houve 5 casos de bacteremia sem foco. Um caso foi de pneumonia associada à ventilação mecânica e um caso de diarreia clostridial.

Não houve complicações mecânicas associadas à ECMO. Em 5 pacientes (33,3%), trombos foram visualizados no sistema, sem necessidade de troca do circuito. Arritmias foram frequentes: 40% dos pacientes apresentaram arritmia supraventricular, sendo a fibrilação atrial a mais comum.

Com respeito à canulação, 4 pacientes apresentaram sangramento, 4 pacientes (26,6%) apresentaram isquemia de membro inferior, necessitando de fasciotomia em 3 (20%), e amputação foi realizada em apenas 1 paciente (6,6%). Uma cânula de perfusão distal foi implantada em todos os pacientes, mas em 5 casos, foi implantada após a canulação devido a complicações técnicas, embora ainda dentro de 24 horas.

Quanto às complicações neurológicas, 2 pacientes sofreram acidente vascular cerebral (um isquêmico e um hemorrágico) e convulsões foram observadas em 2 casos (13,3%). No entanto, a CPC na alta hospitalar foi de 1 ou 2 em 100% dos pacientes.

Nos pacientes que receberam alta hospitalar, o acompanhamento mediano foi de 12 meses (IIQ: 3 a 34), com sobrevida condicional em longo prazo de 100%. Um paciente inicialmente incluído na lista de transplante eletivo após a alta foi submetido a transplante cardíaco de emergência 2 anos após a ECPR. No momento do último acompanhamento, o paciente apresentava boa função do enxerto e nenhuma complicação pós-transplante importante.

## Discussão

Em nosso centro, a utilização de ECMO venoarterial como tratamento para PCR intra-hospitalar tem demonstrado resultados promissores, observando taxas de sobrevida e desfechos neurológicos aceitáveis na alta hospitalar. A ECMO venoarterial serve como uma opção terapêutica eficaz em pacientes cuidadosamente selecionados, particularmente quando as terapias convencionais se mostram insuficientes para alcançar resultados adequados. Além disso, sua aplicabilidade e utilidade se estendem até mesmo a países de baixa e média renda, onde o acesso a outros dispositivos de assistência circulatória pode ser limitado.

A ECPR continua sendo uma terapia pouco comum, mesmo em centros de grande volume. Uma análise observacional de PCR intra-hospitalar em adultos nos Estados Unidos constatou que, entre os anos de 2000 e 2018, menos de 1% dos pacientes com PCR intra-hospitalar relatados foram tratados com ECPR.^23^

Atualmente, não há dados suficientes para identificar os pacientes que poderiam se beneficiar da ECPR. Recomendações internacionais sugerem que cada centro deve estabelecer critérios de inclusão acordados para orientar os médicos no equilíbrio do uso inteligente de recursos entre pacientes considerados com maior probabilidade de sobrevivência após uma PCR.^5^ Embora a coorte do estudo reflita uma população criteriosamente selecionada, as decisões sobre a ECPR foram tomadas de acordo com critérios de inclusão padronizados e por meio de discussão em tempo real dentro da equipe de ECMO. Durante o período do estudo, a disponibilidade de recursos e de pessoal não foram fatores limitantes, sendo todas as decisões sobre a ECPR baseadas na elegibilidade clínica, e não em restrições logísticas.

O tempo de baixo fluxo (a duração entre o início da RCP convencional e o estabelecimento da ECPR) pode ser um dos fatores preditivos de desfechos bem-sucedidos da ECPR.^5,6^ Diretrizes e opiniões de especialistas afirmam que a janela terapêutica ideal para ECPR é de até 60 minutos após a PCR.^24,25^ No entanto, as evidências para esse ponto de corte são baseadas em dados limitados de populações heterogêneas. Estudo recente relatou que o grupo ECPR com duração ≤ 38 minutos apresentou uma incidência significativamente maior de sobrevida até a alta hospitalar em comparação com o grupo ECPR com duração > 38 minutos (40,0% versus 24,7%, p = 0,032). Além disso, a incidência de resultados neurológicos favoráveis na alta hospitalar tendeu a ser maior no grupo ECPR ≤ 38 minutos do que no grupo ECPR > 38 minutos (35,5% versus 24,7%, p = 0,102).^26^

O tempo necessário para estabelecer o suporte ECMO depende muito das capacidades da equipe de ressuscitação e de fatores do paciente. Em nosso centro, a formação de uma equipe multidisciplinar de ECMO nos permitiu padronizar os critérios de inclusão, considerando que a tomada de decisão para ECPR é frequentemente crítica em termos de tempo e influenciada por fatores externos, como a hora do dia e o dia da semana. Portanto, é crucial ter um sistema logístico eficiente em vigor, com pessoal treinado disponível 24 horas por dia, 7 dias por semana, para canulação. Além disso, a equipe de saúde precisa ser capaz de identificar potenciais candidatos à ECPR em até 10 minutos após a PCR, para facilitar a rápida instalação e preparação do equipamento durante emergências.

As taxas de sobrevivência após a ECPR apresentam um amplo espectro, variando de menos de 15% a mais de 50%, embora a maioria dos estudos tenda a convergir em torno de aproximadamente 30%.^27–30^ Num amplo registo nacional de melhoria da qualidade, Girotra et al. referiram uma sobrevivência global até à alta hospitalar de 17% após paragem cardíaca intra-hospitalar tratada com RCP convencional, com melhoria tanto na sobrevivência como nos resultados neurológicos ao longo do tempo.^31^ No entanto, a variação nas taxas de sobrevida entre a RCP convencional e a ECPR provavelmente é influenciada por um potencial viés de seleção, em que pacientes considerados com maior probabilidade de sobreviver têm maior probabilidade de receber ECPR. Comparações pareadas por propensão entre casos de ECPR e RCP convencional revelaram resultados divergentes entre indivíduos que apresentaram PCR intra-hospitalar.^12,30,31^ Em dois estudos distintos com pareamento por propensão, os pesquisadores encontraram dificuldades em parear 50% e 25% dos pacientes submetidos a ECPR com pacientes submetidos a RCP convencional, o que implica diferenças substanciais entre esses dois grupos. Isso pode limitar a aplicabilidade de achados observacionais relacionados à utilização e à sobrevida da ECPR, também podendo indicar obstáculos ao recrutamento de pacientes para ensaios clínicos e à obtenção de equilíbrio de randomização.

Protocolos e algoritmos visam rapidamente identificar casos com maior probabilidade de sobrevida com desfechos neurológicos favoráveis, como pacientes com PCR presenciada em que a RCP de alta qualidade foi iniciada prontamente, bem como aqueles que apresentam condições potencialmente reversíveis, como obstruções coronárias agudas.^5^ Outros fatores que também podem influenciar a indicação de ECPR são idade, causa e momento da PCR, comorbidades e o ritmo cardíaco inicial no início da PCR.^7^

Recentemente, foi publicado o escore de predição de risco chamado RESCUE-IHCA, derivado de 1.075 pacientes. Desses pacientes, 28% sobreviveram até a alta hospitalar, e as seguintes seis variáveis foram identificadas como associadas à mortalidade intra-hospitalar: idade, horário do dia, ritmo inicial, histórico de insuficiência renal, tipo de paciente (cardíaco versus não cardíaco e clínico versus cirúrgico) e duração da PCR.^32^ A maior probabilidade de sucesso é tipicamente observada em pacientes mais jovens (em alguns grupos de trabalho, indivíduos de até 50 anos são considerados para ECPR), com poucas comorbidades, PCR presenciada, preferencialmente durante o dia (quando a logística é mais simples e há maior acesso a pessoal treinado), manobras de RCP imediatas e apropriadas (preferencialmente em unidades de terapia intensiva) e causa cardíaca com ritmo inicialmente desfibrilável.

Nossas taxas de sobrevida são altas e comparáveis às relatadas no registro multicêntrico internacional da ELSO, onde a sobrevida com ECMO foi de 41% e a sobrevida até a alta hospitalar foi de 30% globalmente,^16^ bem como aos achados do estudo RESCUE-IHCA. Além disso, foi documentado desfecho neurológico favorável na maioria dos pacientes.

Os resultados de um ensaio clínico avaliando a ECPR precoce para PCR extra-hospitalar refratária foram publicados. Nesse ensaio multicêntrico, pragmático e randomizado, tanto a ECPR quanto a RCP convencional mostraram efeitos semelhantes na sobrevida, com desfecho neurológico favorável em 30 dias em pacientes que sofreram PCR extra-hospitalar refratária desencadeada por uma arritmia ventricular inicial.^11^ Embora os benefícios potenciais da ECPR em um ambiente adequado possam parecer evidentes, os resultados desse ensaio sugerem que a obtenção de resultados consistentemente superiores não é garantida ao implementar a ECPR de forma pragmática. Isso se aplica mesmo em centros de cirurgia cardíaca onde os profissionais possuem experiência em sua aplicação. As instituições que atualmente oferecem ou consideram a ECPR devem examinar minuciosamente suas considerações logísticas e, posteriormente, avaliar a eficácia do procedimento.

Em relação à PCR extra-hospitalar, três ensaios randomizados (ensaio ARREST, estudo Prague OHCA e ensaio INCEPTION) que abordaram o eventual benefício clínico da ECPR apresentaram resultados divergentes.^9–11^ As evidências derivadas desses três ensaios clínicos randomizados recentes não são contraditórias, mas sim complementares. No primeiro, a ECPR melhorou significativamente a sobrevida, em comparação com a RCP convencional. Em contraste, nos ensaios Prague OHCA e INCEPTION, não houve diferença significativa entre os dois grupos. No entanto, em uma análise secundária do Prague OHCA, excluindo pacientes com RCE pré-hospitalar, a ECPR foi associada a uma melhor sobrevida.^33^

Uma recente metanálise apoia essas evidências favoráveis, mostrando no grupo ECPR uma redução na mortalidade intra-hospitalar, melhores desfechos neurológicos e melhor sobrevida em 30 dias em ambos os cenários: PCR intra-hospitalar e extra-hospitalar.^15^

Embora a ECMO venoarterial não esteja isenta de complicações potenciais, como sangramento, insuficiência renal, infecções e eventos tromboembólicos, a seleção meticulosa de pacientes e o manejo especializado contribuem significativamente para desfechos satisfatórios. Além disso, a ausência de casos relatados de morte cerebral e os favoráveis desfechos neurológicos na alta hospitalar, bem como a ocorrência de apenas dois casos de acidente vascular cerebral em nossa coorte, atestam a segurança e a eficácia geral do procedimento.

O sangramento foi uma das principais complicações em nosso estudo. Comparado ao total de procedimentos de ECMO venoarterial realizados em pacientes com choque cardiogênico, na ECPR, a taxa de sangramento foi menor (46,6% versus 61%).^34^ No entanto, no ramo invasivo do estudo Prague OHCA, o sangramento foi ainda menor (31%), assim como no registro ELSO (21,8%).^10,16^ Neste último caso, provavelmente há subnotificação, uma vez que nem todos os centros que realizam ECMO são relatados; além disso, quantificou apenas o sangramento intestinal, pulmonar, no local da canulação e no local cirúrgico.

Em relação a outras complicações, como acidente vascular cerebral, necessidade de diálise e lesão renal aguda, a taxa foi semelhante à descrita no registro ELSO.^16^ As complicações mais frequentes em nosso estudo, em comparação com esse registro, foram sangramento no local da canulação (26,6% versus 8,3%) e isquemia de membros inferiores (26,6% versus 4,1%). Embora existam publicações que mostram que as complicações vasculares em pacientes submetidos a ECMO aumentam a mortalidade, no registro ELSO, esses pacientes apresentaram sobrevida semelhante à média dos pacientes assistidos por PCR (28% a 35%). Isso provavelmente demonstra que, em nossa população, apesar da maior incidência de complicações vasculares, a sobrevida hospitalar foi igual ou até superior à relatada no registro ELSO (46% versus 30%). A seleção rigorosa de pacientes, com critérios estritos para o manejo da PCR, pode ser um fator explicativo para essa taxa de sobrevida.

A implementação da ECMO venoarterial em nosso centro evidenciou seu potencial para fornecer suporte vital e preencher a lacuna entre eventos cardíacos críticos e a recuperação. Ao oferecer suporte circulatório e respiratório temporário, a ECMO venoarterial permite que os profissionais de saúde estabilizem os pacientes e ganhem tempo precioso para tratar as causas subjacentes da PCR. Uma vantagem notável da ECMO venoarterial reside em sua adequação a ambientes com recursos limitados, onde o acesso a outras tecnologias avançadas de suporte circulatório pode ser restrito. Ao aproveitar os recursos da ECMO venoarterial, as instituições de saúde em países de baixa e média renda podem aprimorar sua capacidade de gerenciar casos cardíacos críticos e, potencialmente, melhorar as taxas de sobrevida dos pacientes.

O presente estudo apresenta limitações inerentes ao seu desenho retrospectivo, bem como à ausência de um grupo controle. Os resultados podem não ser diretamente aplicáveis a outros centros da região, visto que nossa instituição é um centro cardiovascular especializado de alta complexidade, com experiência consolidada em ECMO venoarterial, programa de assistência estruturado e mais de 9 anos de atuação, realizando atualmente mais de 15 implantes por ano. Embora o número de pacientes incluídos na presente série seja limitado, até onde sabemos, ela representa a primeira série sobre ECPR na América Latina. Não incluímos um grupo controle de pacientes tratados apenas com RCP convencional, pois não havia dados abrangentes disponíveis para análise sobre essa população. Isso limita nossa capacidade de estabelecer conclusões comparativas diretas sobre o potencial benefício de sobrevida da ECPR em relação às estratégias padrão de ressuscitação.

## Conclusão

A ECMO venoarterial demonstrou ser uma estratégia viável para o tratamento da PCR intra-hospitalar em nosso centro, oferecendo uma oportunidade de sobrevida e recuperação em pacientes adequadamente selecionados. À medida que evolui a compreensão dessa tecnologia e de sua aplicação, o papel da ECMO venoarterial no tratamento de emergências cardíacas pode ser ainda mais otimizado, beneficiando pacientes tanto em nosso centro quanto em instituições de saúde semelhantes ao redor do mundo.

## Data Availability

Todo o conjunto de dados que dá suporte aos resultados deste estudo está disponível mediante solicitação ao autor correspondente.
