# Supplementary material for: Ressuscitação Cardiopulmonar Extracorpórea para Parada Cardiorrespiratória Intra-Hospitalar: Experiência de um Centro na América Latina
Source: Arq Bras Cardiol. 2026 Apr 14;123(3):e20240820. [Article in Portuguese] doi: 10.36660/abc.20240820 (PMC13128206; doi:10.36660/abc.20240820)
Supplement: Translation

# Translation: Extracorporeal Cardiopulmonary Resuscitation for In-Hospital Cardiac Arrest: A Single-Center Experience in Latin America

**DOI:** 10.36660/abc.20240820i

**Authors:** Lucrecia M. Burgos, Leonardo A. Seoane, Ana Spaccavento, Juan F. Furmento, Juan P. Costabel, Rocio C. Baro Vila, Maria A. de Bortoli, Daniel O. Navia, Fernando Piccinini, Mariano Vrancic, Mirta Diez

**Affiliations:** 1 Buenos Aires Argentina Instituto Cardiovascular de Buenos Aires (ICBA), Heart Failure, Pulmonary Hypertension and Transplant Department, Buenos Aires – Argentina; 2 Buenos Aires Argentina Instituto Cardiovascular de Buenos Aires (ICBA), Critical Care Cardiology Department, Buenos Aires – Argentina; 3 Buenos Aires Argentina Instituto Cardiovascular de Buenos Aires (ICBA), Cardiac Surgery Department, Buenos Aires – Argentina

**Keywords:** Cardiac Arrest, Extracorporeal Membrane Oxygenation, Cardiopulmonary Resuscitation

## Abstract

**Background:**

Extracorporeal cardiopulmonary resuscitation (ECPR) is the use of extracorporeal membrane oxygenation (ECMO) in patients in whom conventional cardiopulmonary resuscitation measures have failed to achieve a sustained return of spontaneous circulation after cardiorespiratory arrest. Limited information is available in centers in Latin America.

**Objective:**

To evaluate in-hospital and long-term clinical outcomes of adult patients treated with ECPR after in-hospital cardiac arrest (IHCA) in a high-complexity center in Argentina.

**Methods:**

A single-center cohort study was performed. Consecutive adult patients who underwent ECPR between 2014 and 2023 were retrospectively analyzed. Witnessed IHCA cases of probable cardiac origin were included.

**Results:**

Fifteen patients were included in the analysis, representing 16% of the venoarterial ECMO cases implanted at the center during this period. The median age was 46 years (interquartile range [IQR]: 34 to 57); 33% were female. Cannulation was peripheral in 93.3%, and 66% of ECMO runs were placed with an initial bridging strategy to recovery. The median duration of circulatory support was 5 days (IQR: 2 to 9). Venoarterial ECMO was successfully removed in 60% of patients, and in-hospital survival was 46%. Median follow-up after discharge was 12 months (IQR: 3 to 34), with 100% long-term survival.

**Conclusion:**

Venoarterial ECMO as a treatment for IHCA in our center demonstrated acceptable survival to hospital discharge and long-term survival. Venoarterial ECMO may be an effective treatment in highly selected patients when conventional therapies fail and may be useful and applicable in a low- and middle-income country with limited access to other circulatory assist devices.

## Introduction

In-hospital cardiac arrest (IHCA) is an acute event that can potentially affect any hospitalized patient. Cardiac arrest (CA) is a major cause of morbidity and mortality worldwide.^1–4^ Despite significant advancements in ventilation administration, medications, chest compressions, and electrical defibrillation over the last 50 years, achieving return of spontaneous circulation (ROSC) with conventional cardiopulmonary resuscitation (CPR) still poses challenges. Moreover, even after ROSC is achieved, some patients may face re-arrest and/or unfavorable neurological outcomes.^5,6^

Extracorporeal cardiopulmonary resuscitation (ECPR) is the utilization of extracorporeal membrane oxygenation (ECMO) in patients in whom conventional CPR measures have failed to achieve sustained ROSC after a CA.^i^ It may be considered when the time without spontaneous circulation is short and resuscitation attempts are adequate (i.e., CPR > 10 minutes, appropriate use of guideline-directed medications such as epinephrine and vasopressin, and defibrillation for shockable rhythms).^1–4^

ECPR is increasingly used worldwide as a rescue technique among patients with refractory CA.^7^ While there are currently no systematic recommendations for the indication of ECMO in this setting, it could be considered as an emergent therapy when conventional CPR fails in selected cases.^8^ Currently, there are no reports of controlled randomized trials comparing the outcomes of ECPR with conventional CPR in IHCA.^4^ In contrast, there are some trials in out-of-hospital cardiac arrest (OHCA) with heterogeneous results.^9–11^

Propensity-matched cohort studies have reported outcomes associated with ECMO use to be superior to those of conventional CPR alone in in-hospital patients with CA.^12–14^ Furthermore, a recent meta-analysis found that ECPR could reduce in-hospital mortality.^15^ However, limited information is available in low- and middle-income countries. Barriers to its application include accessibility, lack of resources, potential complications, and the need for a specialized team for proper implantation and management.

The objective of this study was to analyze and report the characteristics and clinical outcomes of a consecutive cohort of adult patients treated with ECPR after an IHCA at a high-complexity center in Argentina.

## Materials and methods

A single-center cohort study was performed. We reported the characteristics and clinical outcomes of a consecutive cohort of adult patients treated with ECPR after an IHCA at a high-complexity center in Argentina.

### Patients

Consecutive adult patients who underwent ECPR between January 2014 and July 2023 were analyzed. In our hospital, ECPR enrollment criteria included age between 18 and 70 years, witnessed IHCA, and probable cardiac origin (mainly ventricular tachycardia or ventricular fibrillation as the initial rhythm). Absence of previously known life-limiting comorbidities (chronic obstructive pulmonary disease, end-stage renal failure, liver failure, terminal illness), absence of aortic dissection and severe peripheral arterial disease, and estimated time from CA to ECMO flow < 60 minutes were additional criteria for inclusion in this study. Patients who received ECMO support for cardiogenic shock and later experienced CA were excluded from this analysis.

### ECMO team and center description

The ECMO team, although part of the transplant and ventricular assistance clinic, is composed of specialists from various services: cardiovascular surgeons, critical care cardiologists, heart failure specialists, perfusionists, nurses, kinesiologists, cardiologists specializing in ultrasound, anesthesiologists, interventional cardiologists, nutritionists, hematologists, and infectious disease specialists. The ECMO team is a component of the shock team, focused specifically on venoarterial (VA) ECMO as circulatory support. The key team members (cardiovascular surgeons, perfusionists, cardiologists, and nurses) must be available 24 hours a day, 365 days a year, for emergency cannulation or to promptly address ECMO complications. Cannulation-related complications such as vascular injury and limb ischemia were prevented by routine use of distal perfusion catheters. ECMO cannulation was performed by interventional cardiologists or cardiovascular surgeons, depending on the setting.

The primary objectives of establishing an ECMO team were to standardize processes, determine unified criteria for inclusion and exclusion for VA ECMO implantation, and ensure timely ventricular assistance in cases of refractory cardiogenic shock or CA. After implantation, the team is responsible for daily patient monitoring and follow-up, determining the timing and mode of weaning from assistance. The initial focus was on creating checklists for ECMO assembly, purging, and implantation, as well as team activation protocols. Subsequently, protocols were developed for both percutaneous and surgical implantation, anticoagulation management, ultrasound utilization (for implantation, monitoring, and weaning), infection prophylaxis, nutrition, and nursing care.

Establishing the ECMO team, in addition to organizing and standardizing processes, involved theoretical learning and psychophysical skills training.

All core ECMO team members complete a structured institutional training program, including high-fidelity simulation exercises held at least twice a year, aimed at maintaining technical proficiency and team coordination. Since 2021, our center has been certified by the Extracorporeal Life Support Organization (ELSO). According to ELSO data, the mean annual adult VA ECMO volume in certified Latin American centers in 2022 was 5 to 6 runs. ^16^ Currently, our center has an average volume of 15 VA ECMO runs per year.

Our institution applies do not resuscitate protocols under standardized ethical oversight, primarily in patients with end-stage illness or prior advanced directives.

### ECMO support and post-cardiac arrest care management

The ECMO team was activated for patients with refractory CA who did not achieve ROSC after 10 minutes of conventional CPR. In the case of ECMO placement in the catheterization lab, the puncture and cannulation were initiated by interventional cardiologists, whereas in patients located in the intensive care unit, the procedure was led by cardiovascular surgeons. Table 1 outlines the establishment of ECMO support and post-CA care management.

**Table 1:** Establishment of ECMO support and post-cardiac arrest care management

After cannula insertion: – Purge all air from circuit. Check for air or bubbles in circuit, membrane or connections. – Increase RPM and remove clamps on circuit. – VA ECMO blood flow should be ≥3 L/min. Discontinue mechanical compressions. Vasopressor and inotrope infusions, may need to be weaned rapidly to MAP target ≥ 60 mmHg for organ perfusion pressure and < 80 mmHg to minimize risk of left ventricle distension.
Confirm cannula position with fluoroscopy or echocardiography prior to cannula securing and dressing.
MAP measurement with right-sided upper limb arterial line (radial or brachial).
Titrate sweep gas flow mechanical ventilation to avoid hypocarbia and hyperoxia.
Address potential drainage insufficiency with fluids/transfusion/weaning of VA ECMO blood flow if it exceeds requirement; total circulation is made up of native cardiac output, if present, plus VA ECMO blood flow.
Ensure correct placement of endotracheal tube and central venous catheter.
Sedation and analgesia.
Targeted temperature management: 33 to 36 °C for 24 hours.
Perform bedside ultrasound to identify possible complications and assess valve competence and left ventricle distension.

MAP: mean arterial pressure; RPM: revolutions per minute; VA ECMO: venoarterial extracorporeal membrane oxygenation. Adapted from Richardson et al.^8^

External cardiac massage was continued throughout the cannulation phase. We used a modified Seldinger technique to cannulate the femoral vein and femoral artery with cannulas of 19-25 Fr and 15-21 Fr, respectively. ECMO blood flow was adjusted to achieve a target of 2.5 L/min/m^2^, tailored to the patients’ metabolic needs. ECMO gas flow was set to maintain an arterial pCO_2_ of approximately 40 to 45 mm Hg. Heparin infusion was used for anticoagulation to maintain the activated clotting time between 160 and 180 seconds. The target hematocrit was 30% to 35%. An additional 7-9 Fr percutaneous catheter was placed distal to the ECMO arterial cannula in the femoral artery to prevent limb ischemia.^17^ Daily echocardiography was performed. Weaning from ECMO was considered at least 24 to 48 hours after ROSC, following the same protocol in each patient.^18,19^

Central VA ECMO was inserted under general anesthesia using a sternotomy incision, implanting one cannula in the aorta and the other in the superior vena cava.

### Endpoints

The primary endpoint was in-hospital survival. Secondary outcomes included VA ECMO weaning, long-term conditional survival, neurological status at hospital discharge, bleeding and thrombotic events, need for renal replacement, and vascular complications. Neurological status was assessed according to the cerebral performance category (CPC). The performance categories were defined as follows: CPC 1, conscious and alert with normal function or only slight disability; CPC 2, conscious and alert with moderate disability; CPC 3, conscious with severe disability; CPC 4, comatose or in a persistently vegetative state; and CPC 5, certified brain death or dead by traditional criteria. Favorable neurological outcome was defined as a score of 1 or 2.^20^

### Bridge strategy during ECMO support

As part of the center's standard of care and the ECMO team's protocols, when placing VA ECMO, an initial bridge is established, and the mechanical circulatory support (MCS) device is implanted accordingly.^21,22^ For patients who have experienced CA without prior cardiovascular history, the initial bridge is to organ recovery. The bridges and the characteristics that guide their selection are described below:

– Bridge to decision: MCS is indicated to keep the patient alive in cases of acute circulatory collapse refractory to pharmacological treatment with an immediate risk of death until a comprehensive assessment of their condition and therapeutic options can be conducted.
– Bridge to candidacy: Used to improve the function of organs damaged by low output or to improve clinical conditions that increase the risk of transplantation, such as pulmonary hypertension or cardiac cachexia, among others. The use of MCS allows the patient to become a candidate for heart transplantation.
– Bridge to transplant: Indicated as life support for high-risk patients on the waiting list until an organ becomes available.
– Bridge to recovery: The MCS allows the patient to be kept alive until their cardiac function improves sufficiently for explantation. It is used in clinical situations where improvement is presumed, such as acute myocarditis, acute myocardial infarction, peripartum cardiomyopathy, among others.
– Destination therapy: Long-term use of MCS as an alternative to heart transplantation in patients who are not candidates for transplantation.

### Data collection and follow-up

Baseline demographic and clinical data, comorbid conditions, physical examination on admission (heart rate, systolic and diastolic blood pressure), clinical presentation, laboratory findings, previous medical treatment, and therapy implemented for each patient were collected in a dedicated center database. The follow-up was conducted through the review of medical records, and phone contact was established for patients without follow-up.

### Statistical analysis

Continuous variables were expressed as mean and standard deviation or median and interquartile range (IQR), according to the observed distribution. Kolmogorov-Smirnov or Shapiro-Wilk tests were used depending on sample size and distribution characteristics. Categorical variables were expressed as numbers and percentages. SPSS Statistics, version 23.0 (IBM Corporation, Armonk, NY) was used for statistical analysis.

### Ethical considerations

The study was approved by the institutional research and ethics board and was registered on the PRIISA.BA platform of Buenos Aires City Ministry of Health. At the time of hospitalization, patients signed consent for the transfer of personal data for scientific purposes. The study was carried out in accordance with national and international standards for the protection of research subjects, including the Declaration of Helsinki, Resolution of the Ministry of National Health 1480/2011, Ciudad de Buenos Aires law 3301, ANMAT resolution 6677/10, and amendments 4008 and 4009.

## Results

Fifteen patients were included in the analysis, representing 16% of the VA ECMO runs implanted at the center during this period. Forty percent of ECMO procedures were carried out in the catheterization lab, and the rest were performed in the intensive care unit (Central Illustration).

The median age was 46 years (IQR: 34 to 57); 33% were female. No patients had a history of chronic obstructive pulmonary disease, chronic kidney disease, cerebrovascular accident, or anemia. The main underlying etiologies were as follows: acute coronary syndrome (53.3%), electrical storm in arrhythmogenic cardiomyopathy (6.7%), idiopathic cardiomyopathy (13.4%), idiopathic restrictive cardiomyopathy (6.7%), and myocarditis (6.7%). The other baseline characteristics are described in Table 2. One hundred percent of the patients had primary shockable rhythm.

**Table 2:** Baseline characteristics

Variables	n = 15
Age, years, median (IQR)	46 (34-57)
Arterial hypertension	5 (33.3%)
Dyslipidemia	5 (33.3%)
Diabetes	1 (6.7%)
Smoking	2 (13.3%)
Chronic kidney disease	0 (0%)
Atrial fibrillation	2 (13.3%)
Coronary artery disease	1 (6.7%)
Stroke	0 (0%)
Peripheral artery disease	1 (6.7%)
Anemia	0 (0%)
COPD	0 (0%)

COPD: chronic obstructive pulmonary disease; IQR: interquartile range.

Cannulation was peripheral in 93.3%, and 66% of ECMO runs were placed with an initial bridging strategy to recovery. The median low-flow time (from CA to ECMO run) was 41 minutes (IQR: 25 to 57). Regarding left ventricular decompression strategies, 66.6% required intra-aortic balloon pump; 2 patients required surgical venting, and 2 patients underwent percutaneous venting.

The median duration of circulatory support was 5 days (IQR: 2 to 9), and length of stay was 14 days (IQR: 11 to 21).

VA ECMO was successfully removed in 60% of patients, and in-hospital survival was 46% (Figure 1). One patient required an emergency heart transplant.

**Figure 1:** In-hospital and conditional long-term survival.

The most common complications during VA ECMO support were bleeding (46.6%), infections (46.6%), acute kidney injury (46.6%), and prolonged mechanical ventilation requiring tracheostomy (26.6%), as displayed in Table 3.

**Table 3:** Complications of extracorporeal membrane oxygenation runs

Complication	N = 15
Bleeding, n (%)	7 (46.6%)
Acute kidney injury, n (%)	7 (46.6%)
Infections, n (%)	7 (46.6%)
Tracheostomy, n (%)	4 (26.6%)
Arrhythmias, n (%)	6 (40.0%)
Tamponade, n (%)	2 (13.3%)
Lower limb ischemia, n (%)	4 (26.6%)
Stroke, n (%)	2 (13.3%)
Seizures, n (%)	2 (13.3%)
Brain death, n (%)	0 (0%)

The most frequent bleeding was from the cannulation site, occurring in 4 patients (26.6%), followed by tamponade (13.3%). Only one patient presented gastrointestinal bleeding (6.6%). Although renal failure occurred in 46.6% of cases, only 4 patients required hemodialysis (26.6%).

Regarding infections, the most frequently isolated microorganism was *Klebsiella* sp. (13.3%). There were 5 cases of bacteremia without focus. One case was ventilation-associated pneumonia, and one case was clostridial diarrhea.

There were no associated ECMO mechanical complications. Thrombi were visualized in the system, without circuit change in 5 patients (33.3%). Arrhythmias were frequent: 40% of patients presented supraventricular arrhythmia, with atrial fibrillation being the most common.

In relation to cannulation, 4 patients had bleeding, 4 patients (26.6%) lower limb ischemia, requiring fasciotomy in 3 (20%), and amputation in only one patient (6.6%). A distal perfusion cannula was implanted in all patients, but in 5 cases it was performed after cannulation due to technical complications, although still within 24 hours.

Regarding neurological complications, 2 patients had stroke (one ischemic and one hemorrhagic), and seizures were observed in 2 cases (13.3%). However, CPC at discharge was 1 or 2 in 100% of patients.

In patients discharged from the hospital, median follow-up was 12 months (IQR: 3 to 34), with 100% long-term conditional survival. One patient initially placed on the elective transplant list after discharge underwent emergency heart transplantation 2 years after ECPR. At the time of last follow-up, the patient exhibited good graft function and no major post-transplant complications.

## Discussion

In our center, the utilization of VA ECMO as a treatment for IHCA has shown promising results, with acceptable survival rates and neurological outcomes observed at hospital discharge. VA ECMO serves as an effective therapeutic option in carefully selected patients, particularly when conventional therapies prove insufficient in achieving successful outcomes. Moreover, its applicability and usefulness extend even to low- and middle-income countries, where access to other circulatory assist devices may be limited.

ECPR remains an uncommon therapy, even in high-volume centers. An observational analysis of adult IHCA in the United States found that between the years 2000 and 2018, less than 1% of reported IHCA patients were treated with ECPR.^23^

Currently, we lack sufficient data to identify those who could benefit from ECPR. International recommendations suggest that each center should establish agreed-upon inclusion criteria to guide physicians in balancing the intelligent use of resources among patients deemed to have a better likelihood of survival after a CA.^5^ Although the study cohort reflects a highly selected population, ECPR decisions were made according to standardized inclusion criteria and through real-time discussion within the ECMO team. During the study period, resource availability and personnel coverage were not limiting factors, and all ECPR decisions were based on clinical eligibility rather than logistical constraints.

Low-flow time, the duration between the initiation of conventional CPR and the establishment of ECPR, might be one of the predictive factors of successful outcomes of ECPR.^5,6^ Guidelines and expert opinions state that the ideal therapeutic window for ECPR is within 60 minutes of arrest.^24,25^ However, the evidence for this cutoff is based on limited data from heterogeneous populations. A recent study reported that the ECPR group with a duration of ≤ 38 minutes showed a significantly higher incidence of survival to discharge compared to the ECPR group with a duration of > 38 minutes (40.0% versus 24.7%, p = 0.032). Additionally, the incidence of favorable neurological outcomes at discharge tended to be higher in the ECPR ≤ 38 minute group than in the ECPR > 38 minute group (35.5% versus 24.7%, p = 0.102).^26^

The time required to establish ECMO support is highly dependent upon the capabilities of the resuscitation team and patient factors. In our center, the formation of a multidisciplinary ECMO team has enabled us to standardize the inclusion criteria, considering that decision-making for ECPR is often time-critical and influenced by external factors such as the time of day and the day of the week. Therefore, it is crucial to have an efficient logistics system in place with trained personnel available 24/7 for cannulation. Additionally, healthcare staff need to be capable of identifying potential ECPR candidates within 10 minutes of the CA to facilitate rapid setup and priming of the equipment during emergencies.

Survival rates following ECPR exhibit a broad spectrum, ranging from below 15% to over 50%, although most studies tend to converge around approximately 30%.^27–30^ In a large national quality-improvement registry, Girotra et al. reported an overall survival to discharge of 17% after in-hospital cardiac arrest treated with conventional CPR, with both survival and neurologic outcomes improving over time.^31^ Nevertheless, the variation in survival rates between conventional CPR and ECPR is likely influenced by potential selection bias, where patients believed to have a higher chance of survival are more likely to receive ECPR. Propensity-matched comparisons between ECPR and conventional CPR cases have revealed divergent outcomes among individuals experiencing IHCA.^12,30,31^ In two separate propensity-matched studies, researchers encountered difficulties in matching 50% and 25% of ECPR patients with conventional CPR patients, implying substantial differences between these two groups. This could limit the applicability of observational findings related to ECPR utilization and survival, and it might also indicate obstacles to enrolling patients in clinical trials and achieving randomization equipoise.

Protocols and algorithms aim to swiftly identify cases with a higher likelihood of surviving with favorable neurological outcomes, such as patients with witnessed CAs where high-quality CPR was promptly initiated, and those experiencing potentially reversible conditions like acute coronary obstructions.^5^ Other factors that can also influence the indication for ECPR are age, cause of CA, timing, comorbidities, and the initial cardiac rhythm at the onset of the arrest.^7^

Recently, the risk prediction score called RESCUE-IHCA, derived from 1075 patients, was published. Of these patients, 28% survived to discharge, and the following six variables were identified to be associated with in-hospital mortality: age, time of day, initial rhythm, history of renal insufficiency, patient type (cardiac versus non-cardiac and medical versus surgical), and duration of the CA.^32^ The highest probability of success is typically observed in younger patients (in some working groups, individuals up to 50 years old are considered for ECPR), with few comorbidities, a witnessed CA, preferably during the day (when logistics are more straightforward, and there is greater accessibility to trained personnel), immediate and appropriate CPR maneuvers (preferably in critical care units), and a cardiac cause with an initially defibrillable rhythm.

Our observed survival rates are high and comparable to those reported in the international multicenter registry by the ELSO, where ECMO survival was 41% and survival to hospital discharge was 30% globally,^16^ as well as the findings from the RESCUE-IHCA study. Furthermore, a good neurological outcome was documented in most of the patients.

The results of a clinical trial evaluating early ECPR for refractory OHCA were published. In this multicenter, pragmatic, randomized trial, both ECPR and conventional CPR showed similar effects on survival with a favorable neurological outcome at 30 days in patients experiencing refractory OHCA triggered by an initial ventricular arrhythmia.^11^ While the potential benefits of ECPR in a suitable setting may seem apparent, the results of this trial suggest that achieving consistently superior outcomes is not guaranteed when pragmatically implementing ECPR. This holds true even in cardiosurgical centers where providers possess experience in its application. Institutions currently offering or contemplating ECPR should thoroughly examine their logistical considerations and subsequently assess the procedure's efficacy.

In OHCA, three randomized trials (ARREST trial, Prague OHCA study, and INCEPTION trial) addressing the eventual clinical benefit of ECPR had diverging results.^9–11^ The evidence derived from these three recent randomized controlled trials is not contradictory but rather complementary. In the first one, ECPR significantly improved survival, compared to conventional CPR. In contrast, in the Prague OHCA and INCEPTION trials, there was no significant difference between both groups. However, in a secondary analysis of Prague OHCA, excluding patients with prehospital ROSC, ECPR was associated with improved survival.^33^

A recent meta-analysis supports this favorable evidence, showing in the ECPR group a reduction in in-hospital mortality, improved neurological outcomes, and improved 30-day survival in both scenarios: IHCA and OHCA.^15^

While VA ECMO is not without potential complications such as bleeding, renal failure, infections, and thromboembolic events, meticulous patient selection and expert management significantly contribute to successful outcomes. Moreover, the absence of reported brain death cases and good neurological outcomes at discharge and the occurrence of only two strokes in our cohort attest to the procedure's overall safety and effectiveness.

Bleeding was one of the main complications in our study. Compared to our total VA ECMO runs assisted by cardiogenic shock, in ECPR the bleeding rate was lower (46.6% versus 61%).^34^ However, in the invasive branch of the Prague OHCA trial, bleeding was even lower (31%), as was the case in the ELSO Registry (21.8%).^10,16^ In the latter case, there is probably underreporting, since not all centers that perform ECMO are reported, in addition to quantifying only intestinal, pulmonary, cannulation site, and surgical site bleeding.

In relation to other complications, such as stroke, need for dialysis, and acute kidney injury, the rate was similar to those described in the ELSO Registry.^16^ The complications that were most frequent in our study, in relation to this registry, were bleeding from the cannulation site (26.6% versus 8.3%) and lower limb ischemia (26.6% versus 4.1%). Although there are publications that show that vascular complications in ECMO patients increase mortality, in the ELSO Registry, those patients had similar survival to the average of patients assisted in CA (28% to 35%). This probably demonstrates that in our population, even with greater vascular complications, in-hospital survival has been equal to, or even greater than that reported in the ELSO Registry (46% versus 30%). The strict selection, with rigorous criteria of patients to manage in CA, may be a reason that explains this survival rate.

The implementation of VA ECMO in our center has highlighted its potential to provide life-saving support and bridge the gap between critical cardiac events and eventual recovery. By offering temporary circulatory and respiratory assistance, VA ECMO allows healthcare providers to stabilize patients and buy precious time to address the underlying causes of CA. One notable advantage of VA ECMO lies in its suitability for resource-constrained settings, where access to other advanced circulatory support technologies might be limited. By leveraging VA ECMO's capabilities, healthcare facilities in low- and middle-income countries can enhance their ability to manage critical cardiac cases and potentially improve patient survival rates.

This study has certain limitations inherent in its retrospective design, as well as the absence of a control group. These results may not be directly applicable to other centers in the region, since our institution is a specialized high-complexity cardiovascular center, renowned for VA ECMO, with a well-established assistance program and over 9 years of experience, currently performing more than 15 implants annually. Although the number of patients included in this series is limited, to the best of our knowledge it represents the first series on ECPR in Latin America. We did not include a control group of patients treated with conventional CPR alone, as comprehensive data on this population were not available for analysis. This limits our ability to draw direct comparative conclusions regarding the potential survival benefit of ECPR over standard resuscitation strategies.

## Conclusion

VA ECMO has demonstrated its value as a viable option for treating IHCA in our center, offering an opportunity for survival and recovery in appropriately selected patients. As our understanding of this technology and its application continues to evolve, VA ECMO's role in managing cardiac emergencies can be further optimized, benefiting patients both in our center and in similar healthcare settings worldwide.

## Data Availability

All datasets supporting the results of this study are available upon request from the corresponding author.
